# Pandemic Stress and Its Correlates among Pregnant Women during the Second Wave of COVID-19 in Poland

**DOI:** 10.3390/ijerph182111140

**Published:** 2021-10-23

**Authors:** Michalina Ilska, Anna Kołodziej-Zaleska, Anna Brandt-Salmeri, Heidi Preis, Marci Lobel

**Affiliations:** 1 Institute of Psychology, University of Silesia in Katowice, Grażyńskiego Street 53, 40-126 Katowice, Poland; anna.kolodziej-zaleska@us.edu.pl (A.K.-Z.); anna.brandt-salmeri@us.edu.pl (A.B.-S.); 2 Department of Psychology, Stony Brook University, Stony Brook, NY 11794, USA; heidi.preis@stonybrook.edu (H.P.); marci.lobel@stonybrook.edu (M.L.)

**Keywords:** pregnancy, COVID-19, pandemic stress, correlates of stress, infection, preparedness

## Abstract

**Background:** The ongoing COVID-19 pandemic has created numerous stressful conditions, especially for vulnerable populations such as pregnant women. Pandemic-related pregnancy stress consists of two dimensions: stress associated with feeling unprepared for birth due to the pandemic (Preparedness Stress), and stress related to fears of perinatal COVID-19 infection (Perinatal Infection Stress). The purpose of our study was to elucidate the association between various factors—sociodemographic, obstetric, pandemic-related, and situational—and pandemic stress in its two dimensions during the second wave of the COVID-19 pandemic in Polish pregnant women. **Methods:** A cross-sectional study with a total of 1119 pregnant women recruited during the second wave of the COVID-19 pandemic in Poland (between November 2020 and January 2021). Participants were recruited via social media to complete an online study questionnaire that included sociodemographic, obstetric, situational, and COVID-19 pandemic factors, as well as the Pandemic-Related Pregnancy Stress Scale (PREPS). **Results:** Nearly 38.5% of participants reported high Preparedness Stress; 26% reported high Perinatal Infection Stress. Multivariate analyses indicated that lack of COVID-19 diagnosis, higher compliance with safety rules and restrictions, and limited access to outdoor space were independently associated with moderate to severe levels of Infection Stress. Current emotional or psychiatric problems, nulliparity, limited access to outdoor space, and alterations to obstetric visits were independently associated with moderate to severe Preparedness Stress. **Conclusion:** Study findings suggest that particular attention should be focused on the groups of pregnant women who are most vulnerable to pandemic-related stress and therefore may be more prone to adverse outcomes associated with prenatal stress.

## 1. Introduction

The 2019 coronavirus disease (COVID-19) pandemic is a global health threat, and by far the largest outbreak of an infectious illness in modern history. The COVID-19 pandemic constitutes a significant source of distress for all people but may be particularly stressful for vulnerable groups [1]. Pregnant women are a high-risk population due to the potential dual impact on mother and fetus [2]. Pregnancy is a particularly critical period for women’s mental health [3,4]. Depression and anxiety are some of the most prevalent pregnancy morbidities that have increased since the start of the COVID-19 pandemic [5,6,7]. Tomfohr-Madsen et al. [5] observed higher anxiety prevalence in pregnant women, which is potentially linked to exposure to pandemic chronic stressors and ongoing uncertainty. These authors point out that rates of antenatal depression and anxiety are significantly elevated during the COVID-19 pandemic compared to historical pre-pandemic norms, for example [8]. Other reports on the mental health of pregnant women during the pandemic confirm these trends. Researchers highlight the prevalence of anxiety symptoms [2,9,10], severe pandemic stress [9,11,12], and depression [2,13,14] and also indicate that expectant and postpartum women have higher levels of anxiety and depression compared to similar cohorts assessed before the outbreak [15,16,17,18]. Studies of the determinants of anxiety and depression in pregnancy conducted during the COVID-19 pandemic have confirmed the importance of risk factors described previously as well as stressors related to pandemic circumstances [19,20].

The COVID-19 pandemic is a danger to reproductive and perinatal health both directly, through infection itself, and indirectly, as a consequence of changes in health care, social policy, and social and economic circumstances [21]. The pandemic has introduced widespread chronic fear of infection and, in pregnant women, fear for the health of the fetus in the face of the spreading virus [11,22]. Pandemic stress as a consequence of these circumstances includes infection stress and stress related to preparing for childbirth [22]. Previous studies indicate that prenatal stress (including pandemic stress) and fear of childbirth are factors that may disrupt preparation for delivery and the course of the delivery itself and increase the likelihood of adverse birth outcomes including low birth weight and preterm delivery [23,24,25]. Moreover, recent research indicates that pandemic-related stress is a powerful construct that can affect the mental health of pregnant women, including an increase in symptoms of depression and anxiety [9,20].

A growing body of evidence confirms the harmful consequences of COVID-19 for perinatal physical and mental health. A review carried out by Chmielewska et al. [15] showed that global maternal and fetal outcomes have worsened during the COVID-19 pandemic, with an increase in maternal deaths, stillbirths, ruptured ectopic pregnancies, and maternal depression. Pregnant women are among those who are most worried and concerned about spreading or becoming infected by SARS-CoV-2 [26,27]. Numerous factors may have intensified the worries of pregnant women, including the diverse range of symptoms and complications caused by the disease, limited scientific knowledge about its impact on fetal well-being, confinement, changes in daily routine, transformations of social life, financial problems, and interruptions of prenatal care [2,28].

The pandemic unfolded in a wave-like manner. The outbreak of COVID-19 in Poland began at the end of March 2020 and reached its first peak during March and April 2020, with few infections and a small number of deaths. The first lockdown was introduced at this time as a preventive measure, which limited the possibility of movement, medical care (canceled or rescheduled medical appointments, introduction of telephone consultations), and the functioning of maternity wards and delivery rooms (suspension of appointments, births without a companion) [29,30]. The following months saw a slow return to normalcy, along with the re-opening of the economy, and a gradual lifting of restrictions in maternity care (e.g., accompanied births resumed, and a less restrictive protocol was adopted for the treatment of mothers infected with the virus).

The second wave of the pandemic in Poland, which started in November 2020 and lasted until January 2021, differed from the first in many respects. A sharp increase was observed in incidence of the coronavirus, with numerous deaths and hospitalizations of people suffering from severe COVID-19. A second lockdown was introduced along with shutdown of the economy, schools, and the return of restrictions relating to travel [31]. Constraints in medical care were reintroduced, including maternity care (suspension of accompanied labor, uncertainty about place of delivery, and total ban on hospital visitors). The second wave was also accompanied by changes in the public mood. Although the second wave was objectively more threatening than the first, there was less adherence to preventive measures. Research conducted by Chodkiewicz and colleagues [32] suggests that after initial mobilization in the first wave, stress became chronic and resilience mechanisms were increasingly ineffective, leading to psychological burnout. Additional studies have documented fatigue, burnout, loneliness, and a rise in anxiety, depression, and post-traumatic stress disorder [33,34,35,36].

Therefore, the aim of this study was to investigate the magnitude of pandemic stress in pregnant women during the second wave of the pandemic in Poland and identify its sociodemographic, obstetric, and situational correlates, including pandemic conditions.

## 2. Materials and Methods

From November 2020 to January 2021, we recruited a sample of 1119 pregnant women through social media (i.e., Facebook, pregnancy and birth forums, the Polish Childbirth with Dignity Foundation). A cross-sectional study design with non-random sampling was used. Research assistants posted study advertisements on pregnancy-related social media that directed women to a link with the study questionnaire. The online questionnaire was completed through LimeSurvey, an online survey system. Inclusion criteria were Polish speaking women over 18 years of age who were pregnant during completion of the survey. The research procedure was approved by the Ethics Committee of Silesian University in Katowice (KEUS.43/05.2020).

### 2.1. Methods

COVID-19-related stress. The Pandemic-Related Pregnancy Stress Scale (PREPS [22]; Polish adaptation [11]) is a novel instrument that assesses prenatal stress during the pandemic. The PREPS has been translated into several languages and has been found to have good psychometric properties in different populations [12,19,20]. The PREPS includes a subscale that assesses stress related to preparation for birth and the postpartum period due to the pandemic (PREPS-Preparedness; PREPS-PS) and a second subscale that assesses stress involving concerns about infection of oneself or one’s fetus/baby (PREPS-Infection; PREPS-IS). Both scales were internally consistent (PREPS-PS α = 0.83; PREPS-IS α = 0.79). A third PREPS subscale assessing positive appraisal was not pertinent to this study and therefore not used. Scores for each PREPS scale are calculated as mean item response on a scale from 1 = Very little to 5 = Very much.

Sociodemographic characteristics included maternal age (coded younger < 35/older ≥ 35), financial status (below average/average/above average), relationship status (some or no relationship/married or cohabiting), and level of education (high school/bachelor/postgrad).

Obstetric factors included unplanned pregnancy (no/yes), nullipara (no/yes), gestational age (in weeks and coded by trimester), high-risk pregnancy (no/yes/unsure), chronic medical conditions (no/yes), fertility treatments (no/yes), and length of time trying to conceive (up to a year/one year or more).

Situational predictors. Four factors were assessed with dichotomous questions (no/yes): experience of lifetime abuse, current emotional or psychiatric problems, major life events while pregnant, and feelings of discrimination or harassment because of race, sexuality, gender, or body size.

COVID-19-related conditions included loss of income because of COVID-19 (no/yes), COVID-19 tests in the last 2 months (no/yes), COVID-19 diagnosis in the last 2 months (no/yes), suspected COVID-19 infection without being medically diagnosed (no/yes/unsure), obstetric visit canceled or rescheduled because of COVID-19 (no/yes), telemedicine (no/ yes, but only during COVID/yes, in the past), access to outdoor space (yes, whenever I want/sometimes/rarely), and compliance with safety rules and restrictions (not much or a little/average or a lot).

### 2.2. Statistical Analysis

Mean differences in the continuous PREPS-IS and PREPS-PS stress score for women with different sociodemographic characteristics, obstetric factors, situational factors, and COVID-19-related conditions were evaluated using Independent Sample *t*-tests or ANOVA as appropriate. Following these steps, all variables that exhibited significant associations with the continuous PREPS-IS and PREPS-PS stress score in bivariate analyses were entered into a binary logistic regression model to calculate unadjusted and adjusted odds for high levels of PREPS-IS and PREPS-PS. Cut-off scores (≥4 on the 1–5 response scale) were used to identify women experiencing moderate or severe levels of stress [37]. The criterion for statistical significance was *p* < 0.05 for all analyses.

## 3. Results

Participants were on average 29.79 ± 3.81 years old, with an average gestational age of 25 weeks (25.43 ± 9.73). Almost half of the participants were nulliparas (*n* = 494, 44.1%). Sixty-three women (5.6%) reported being diagnosed with COVID-19 during pregnancy, and one-quarter (*n* = 253, 22.6%) thought they might have contracted COVID-19 during pregnancy but were not diagnosed. Other participant characteristics are displayed in Table 1.

Approximately a quarter (26.1%) and more than a third (38.5%) of the women scored a 4 or higher on the PREPS-IS subscale and PREPS-PS subscale, respectively, indicating high levels of COVID-19-related pregnancy stress.

We investigated the association of PREPS factors with sociodemographic variables (age, relationship status, financial status, education), obstetric characteristics (unplanned pregnancy, nullipara, trimester, high-risk pregnancy, chronic medical conditions, fertility treatment, length of time trying to conceive), and situational factors (lifetime abuse, current emotional or psychiatric problems, major life event while pregnant, discrimination) (Table 1). We also examined associations with COVID-19-related conditions (income lost, COVID-19 test, diagnosis and perceived risk of COVID-19, prenatal care appointment alteration, telemedicine during COVID-19, access to outdoor space, safety rule restrictions) (Table 2).

In the bivariate analyses, PREPS-IS was related to some of the obstetric factors, namely, high-risk pregnancy and length of time trying to conceive. PREPS-IS was also associated with current emotional or psychiatric problems and discrimination (see Table 1). As shown in Table 2, PREPS-IS was also associated with all but one of the COVID-19-related variables. The omnibus F-test was significant for telemedicine during pregnancy; however, the post hoc analysis showed no significant differences.

PREPS-PS was associated with financial status, nullipara, and length of time trying to conceive. PREPS-PS was also related to current emotional or psychiatric problems, major life events during pregnancy, and discrimination (see Table 1). As shown in Table 2, PREPS-PS was also associated with all but one of the COVID-19-related variables (see Table 2). The omnibus F-test was significant for suspected COVID-19 infection; however, the post hoc analysis showed no significant differences. Two logistic regression analyses were carried out to calculate the adjusted odds ratio (AOR) for those who reported the highest level of PREPS-IS and PREPS-PS. As shown in Table 3, the model predicting high levels of Perinatal Infection Stress incorporated variables that exhibited significant bivariate associations with a continuous PREPS-IS score. This regression model predicted 4% of the variance in PREPS-IS, with COVID-19 diagnosis (AOR 4.02, *p* < 0.01), compliance with the safety rules and restrictions (AOR 3.05, *p* < 0.01), and limited access to outdoor space (AOR 1.49, *p* < 0.05), uniquely increasing the odds of high perinatal infection stress.

As shown in Table 4, the model predicting high levels of Preparedness Stress incorporated variables that exhibited significant bivariate associations with the continuous PREPS-PS score. The regression model included sociodemographic, obstetric, situational, and COVID-19-related variables, which predicted 8% of the variance in PREPS-PS, with nulliparity (AOR 1.51, *p* < 0.05), current emotional or psychiatric problems (AOR 1.52, *p* < 0.05), income lost because of COVID-19 (AOR 1.36, *p* < 0.05), obstetric visits canceled or rescheduled (AOR 1.52, *p* < 0.05), and limited access to outdoor space (AOR 2.21, *p* < 0.001) uniquely increasing the odds of high perinatal Preparedness Stress.

## 4. Discussion

The COVID-19 pandemic has pervasive consequences for society including death, economic uncertainty, and strained health care systems. Moreover, the pandemic has triggered a wide variety of psychiatric problems, including anxiety and depression, especially in sensitive populations such as pregnant women [5,38,39]. Additional factors related to pandemic conditions and resulting pandemic stress also threaten maternal mental health.

The current study identified the magnitude and correlates of pandemic-related pregnancy stress during the second wave of COVID-19 in Poland. Nearly a third of pregnant women experienced elevated levels of stress related to feeling unprepared for birth or being worried about perinatal infection. The present research is consistent with other studies carried out in Poland, including those devoted to the COVID-19 pandemic’s negative impact on various dimensions of mental health in pregnant women [40,41].

Sociodemographic, obstetric, and situational factors including pandemic conditions were important correlates of this stress. Most of these factors were specific to one of the two dimensions of pandemic-related prenatal stress, but some—in particular, the pandemic conditions—were associated with both stress about perinatal infection and about feeling unprepared for birth. These common pandemic-related correlates of stress included uncertainty about being ill with COVID-19, limited access to outdoor space, cancelation or postponement of obstetric appointments, and compliance with safety rules and restrictions. Similarly, trying to conceive for more than a year, as well as feeling discriminated against and experiencing emotional and psychiatric problems, were associated with higher levels of pandemic stress of both types.

Although a more limited number of factors distinguished women who were experiencing moderate or severe levels of stress, pandemic conditions were the only factors associated with moderate or severe infection stress; similarly, pandemic conditions constituted a majority of the factors associated with moderate or severe birth preparation stress. Notably, limited access to outdoor space was the only pandemic-related factor significantly associated with high levels of both types of stress. These results parallel those of comparable studies conducted in the US, Germany, and Switzerland during the first wave of the pandemic [9,12]. It is instructive that alterations of obstetric appointments were associated with maternal stress, for the pandemic disrupted normal ways of preparing for childbirth, including the regularity of obstetric appointments according to an established schedule, the availability of medical care in situations that threaten the health of the mother or baby, and participation in antenatal classes. Research from the first wave of the pandemic in Poland also showed that prenatal care appointment cancelation or rescheduling was associated with pandemic stress in pregnant women [10]. The availability, stability, and continuity of medical care during pregnancy are crucial for a sense of security in pregnancy. The pandemic has highlighted pre-existing challenges related to the delivery of standard, high quality, and accessible prenatal care in Poland [42,43]. These findings reinforce the urgent need to prioritize safe, accessible, and equitable maternity care within the strategic response to this pandemic, and in future health crises.

Study findings also suggest that during the pandemic, close attention should be focused on particular groups of pregnant women, similarly identified by prior research as vulnerable to high maternal stress [12,23,37]: women pregnant for the first time, those with a high-risk pregnancy, women who have been trying to conceive for a long time, women who feel discriminated against for various reasons, those who have experienced major life events during pregnancy, and those with other emotional and psychiatric difficulties. These groups experience a higher level of pandemic-related pregnancy stress and therefore may be more prone to complications associated with prenatal stress, including preterm birth, low birthweight, and other outcomes that are well-recognized consequences of high maternal stress during pregnancy [44,45]. For these women, early intervention and the provision of psychological support tailored to their needs may also prevent the development or aggravation of psychopathology.

A higher level of pandemic-related pregnancy stress was also associated with women’s sense of uncertainty around contracting COVID-19. It should be noted that during the second wave of the pandemic in Poland, there was very limited availability of tests, and thus individuals had little knowledge about their possible SARS-CoV-2 infection. A small percentage of women were tested for SARS-CoV-2, and the percentage of pregnant women who knew they had already had COVID-19 was also low. However, almost one-third suspected that they had contracted COVID-19. These women experienced higher pandemic stress of both types: related to infection fear and to lack of preparation for birth. Thus, increasing access to testing would likely help alleviate maternal stress. Research reports that appeared during this time showed that having COVID-19 provides basic immunity against recurrence and reduces the risk of serious complications in the event of another infection [46,47]. This message was widespread in the media and online and is the likely reason why women who reported a prior infection experienced lower stress. Moreover, a stress exposure mechanism may also be at play, reflecting confidence about the ability to manage stress related to the virus among those who were ill and recovered [48].

Interestingly, we found that greater compliance with safety rules and restrictions was associated with higher pandemic-related stress. In other studies, higher anxiety related to COVID-19 has been associated with a tendency to comply with safety rules during the pandemic [49], or with undertaking various protective behaviors [50]. The association that we uncovered between compliance and stress may thus reflect greater cautiousness among pregnant women harboring fears and concerns about infection and birth. However, it is also possible that vigilance with recommended activities designed for safety and health may reinforce or activate fears related to the pandemic and thus intensify pandemic-related stress [48,51]. More in-depth, longitudinal research may be able to untangle these possibilities and distinguish levels of compliance that are healthy and protective from hypervigilance or extreme behaviors that suggest underlying pathology.

### 4.1. Implications for Practice and/or Policy

Given the pandemic context and the vulnerability of pregnant women, it is imperative to recognize distress signals in order to prevent the development or aggravation of psychopathology. Such observations should be made continuously, at various stages of the pandemic, making it possible to understand the dynamics of these changes and respond with adequate interventions, tailoring support to specific needs.

### 4.2. Limitations and Strengths

One of the limitations of this study is the recruitment method, which excluded women who had no access to the internet or social media. As a consequence, the results may not be widely generalizable. Another limitation is the cross-sectional nature of the research, which prevents us from ascertaining whether study variables are predictors or consequences of pandemic-related pregnancy stress. Some may have bidirectional associations with stress. Furthermore, because data were collected exclusively by self-report, we cannot confirm their accuracy.

Another study limitation stems from the online recruitment method, which can introduce bias into the sample. During the pandemic, conducting face-to-face research was difficult or impossible. Future research should consider interview-based assessments and medical chart data to replicate and extend these findings.

Nevertheless, this research also possesses a number of strengths. The use of a well-validated instrument to assess pandemic-related stress and its correlates in a large sample of women pregnant during a time of national emergency provides critical information that can be used to protect the health of childbearing women and their offspring, and these data offer a foundation to examine longer-term effects of the pandemic on this vulnerable population.

## 5. Conclusions

The COVID-19 pandemic and its multiple waves have created numerous conditions that generate stress for pregnant women related to the possibility of infection of themselves or their baby, and stress related to their preparation for childbirth. This study contributes to our understanding of pregnant women’s experiences during an especially dangerous period of the COVID-19 pandemic in Poland and extends the literature on stress during pregnancy. Findings highlight which women are at the greatest risk of elevated stress and offer insight into how this stress might be reduced.

## Figures and Tables

**Table 1:** Sample characteristics and mean differences in PREPS-IS and PREPS-PS scale score based on sociodemographic characteristics, obstetric factors, and other predictors (*N* = 1119).

Sociodemographic Characteristics	*N* (%)	PREPS-IS	PREPS-PS
Age (years)		t = −0.18	t = −0.55
Younger (< 35)	986 (88.1)	3.11 ± 1.04	3.55 ± 0.88
Older ( ≥ 35)	133 (11.9)	3.13 ± 0.96	3.51 ± 0.77
Relationship status		t = −0.02	t = −0.86
Some or no relationship	38 (3.4)	3.12 ± 1.01	3.67 ± 0.98
Married or cohabiting	1077 (96.2)	3.12 ± 1.03	3.54 ± 0.86
Financial status		F = 0.51	F = 8.69 ***
Below average	65 (5.8)	3.03 ± 1.10	3.60 ± 0.90 ^a,b^
Average	701 (62.6)	3.14 ± 1.03	3.62 ± 0.85 ^a^
Above average	353 (31.5)	3.09 ± 1.00	3.39 ± 0.86 ^b^
Education		F = 1.04	F = 0.29
High school	129 (11.5)	3.03 ± 1.13	3.56 ± 0.97
Bachelor	108 (9.7)	3.03 ± 1.09	3.61 ± 0.86
Postgrad	882 (78.8)	3.14 ± 1.00	3.54 ± 0.85
**Obstetric Factors**	***N* (%)**	**PREPS-IS**	**PREPS-PS**
Unplanned pregnancy		t = 0.47	t = 0−1.18
Yes	894 (79.9)	3.12 ± 1.02	3.58 ± 0.86
No	225 (20.1)	3.09 ± 1.07	3.61 ± 0.87
Nullipara		t = −0.87	t = 2.57 *
Yes	494 (44.1)	3.14 ± 1.02	3.62 ± 0.89
No	614 (54.9)	3.11 ± 1.02	3.48 ± 083
Trimester		F = 0.52	F = 2.74
1st	181 (16.2)	3.18 ± 1.00	3.42 ± 0.87
2nd	384 (34.3)	3.11 ± 1.06	3.60 ± 0.86
3rd	554 (49.5)	3.09 ± 1.02	3.55 ± 0.86
High-risk pregnancy		F = 4.03 *	F = 2.62
Yes	127 (11.3)	3.32 ± 1.00 ^a^	3.68 ± 0.83
No	934 (83.5)	3.08 ± 1.03 ^b^	3.52 ± 0.87
Unsure	58 (5.2)	3.29 ± 1.05 ^a,b^	3.70 ± 0.84
Chronic medical conditions		F = 1.26	F = 1.71
Yes	332 (29.7)	3.19 ± 1.03	3.62 ± 0.85
No	779 (69.6)	3.08 ± 1.02	3.52 ± 0.87
Unsure	8 (0.7)	3.05 ± 1.38	3.66 ± 1.04
Fertility treatments		t = −0.19	t = 0.91
Yes	64 (5.7)	3.14 ± 1.04	3.55 ± 0.86
No	1055 (94.2)	3.11 ± 1.03	3.45 ± 0.91
Length of time trying to conceive		t = −2.38 *	t = −2.84 **
Up to a year	987 (88.2)	3.09 ± 1.04	3.52 ± 0.87
One year or more	132 (11.8)	3.30 ± 0.94	3.75 ± 0.79
**Situational Predictors**	***N* (%)**	**PREPS-IS**	**PREPS-PS**
Lifetime abuse		t = −0.99	t = −0.25
Yes	59 (5.3)	2.98 ± 1.05	3.52 ± 0.89
No	1060 (94.7)	3.12 ± 1.03	3.55 ± 0.86
Current emotional or psychiatric problems		t = 2.08 *	t = 4.11 ***
Yes	194 (10.4)	3.25 ± 1.01	3.78 ± 0.81
No	925 (89.6)	3.09 ± 1.03	3.50 ± 0.87
Major life event while pregnant		t = 0.62	t = 2.82 **
Yes	282 (25.2)	3.15 ± 1.03	3.67 ± 0.81
No	837 (74.8)	3.10 ± 1.03	3.51 ± 0.88
Felt discriminated against		t = 2.03 *	t = 3.73 ***
Yes	53 (4.7)	3.38 ± 0.96	3.98 ± 0.74
No	1066 (95.3)	3.10 ± 1.03	3.53 ± 0.86

Note: * *p* < 0.05; ** *p* < 0.01; *** *p* < 0.001. Means with different superscripts are significantly different at *p* < 0.05 in a post hoc Scheffé test.

**Table 2:** Sample characteristics and mean differences in PREPS-IS and PREPS-PS scale score based on COVID-19-related conditions (*N* = 1119).

COVID-19-Related Conditions	*N* (%)	PREPS-IS	PREPS-PS
Loss of income because of COVID-19		t = −0.31	t = −2.77 **
Yes	259 (23.1)	3.13 ± 1.08	3.68 ± 0.87
No	860 (76.9)	3.11 ± 1.01	3.51 ± 0.86
COVID-19 test		t = −1.73	t = 0.78
Yes	165 (14.7)	2.99 ± 0.95	3.58 ± 0.91
No	954 (85.3)	3.14 ± 1.04	3.54 ± 0.86
COVID-19 diagnosis		t = −2.61 **	t = −0.83
Yes	63 (5.6)	2.79 ± 0.84	3.46 ± 0.89
No	1056 (94.4)	3.13 ± 1.03	3.55 ± 0.86
Suspected COVID-19 infection		F = 4.11 *	F = 3.45 *
Yes	148 (13.2)	2.93 ± 1.01 ^a^	3.62 ± 0.90
No	718 (64.2)	3.11 ± 1.04 ^a,b^	3.50 ± 0.87
Unsure	253 (22.6)	3.23 ± 1.00 ^b^	3.65 ± 0.80
Obstetric visit lost or rescheduled		t = −2.74 **	t = −2.84 **
Yes	161 (14.4)	3.32 ± 0.98	3.82 ± 0.72
No	958 (85.6)	3.08 ± 1.03	3.50 ± 0.88
Telemedicine		F = 3.04 *	F = 5.04 **
No	859 (76.8)	3.07 ± 1.04	3.51 ± 0.88 ^a^
Yes, but only during COVID	223 (19.9)	3.23 ± 1.00	3.71 ± 0.80 ^b^
Yes, in the past	37 (3.3)	3.34 ± 0.77	3.44 ± 0.85 ^a,b^
Access to outdoor space		F = 7.59 **	F = 11.04 ***
Yes, whenever I want	945 (84.5)	3.06 ± 1.03 ^a^	3.50 ± 0.86 ^a^
Sometimes	142 (12.7)	3.41 ± 0.95 ^b^	3.84 ± 0.81 ^b^
Rarely	32 (2.9)	3.32 ± 1.18 ^a,b^	3.81 ± 0.88 ^a,b^
Compliance with safety rules and restrictions		t = 6.71 ***	t = 2.11 *
not much or a little	72 (6.4)	2.34 ± 1.10	3.29 ± 1.06
average or a lot	1047 (93.6)	3.17 ± 1.00	3.57 ± 0.85

Note: * *p* < 0.05; ** *p* < 0.01; *** *p* < 0.001; Means with different superscripts are significantly different at *p* < 0.05 in a post hoc Scheffé test.

**Table 3:** Binary multivariate logistic regression predicting high levels of Infection Stress—PREPS-IS (*N* = 1119).

	PREPS-IS	
	AOR	95% CI
**Obstetric factors**		
High-risk ^†^	1.3	0.87, 1.96
Length of time trying to conceive	1.09	0.72, 1.65
**Situational factors**		
Emotional or psychiatric problems	1.15	0.81, 1.64
Discrimination	1.14	0.62, 2.11
**COVID-19-related factors**		
No COVID diagnosis	4.02 **	1.69, 9.56
Perceived risk of having had COVID-19 ^†^	1.06	0.79, 1.41
Appointment altered	1.35	0.91, 1.99
Telemedicine obstetrician ^†^	1.19	0.84, 1.67
Limited access to outdoor space ^†^	1.49 *	1.04, 2.1
Compliance with safety rules and restrictions	3.05 **	1.44, 6.48
	*R^2^* = 0.04	

* *p* < 0.05, ** *p* < 0.01. AOR—Adjusted Odds Ratio; CI—Confidence Interval. ^†^ Women who reported being high-risk and those who were unsure were grouped together. Women who reported perceived risk of having COVID-19 and those who were unsure were grouped together. Women who reported no telemedicine obstetrician during COVID-19 and those who reported it before the pandemic were grouped together. Women who reported sometimes or rarely having access to outdoor space were grouped together.

**Table 4:** Binary multivariate logistic regression predicting high levels of Preparedness Stress (*N* = 1119).

	PREPS-PS	
	AOR	95% CI
**Sociodemographic factors**		
Financial status ^†^	0.93	0.54, 1.62
**Obstetric factors**		
Nulliparity	1.51 **	1.17, 1.95
Length of time trying to conceive	1.21	0.82, 1.78
**Situational factors**		
Discrimination	1.63	0.91, 2.94
Emotional or psychiatric problems	1.52 *	1.09, 2.14
Major life event	1.12	0.84, 1.51
**COVID-19-related factors**		
Income lost	1.36 *	1.01, 1.83
Perceived risk of having had COVID-19 ^†^	1.17	0.90, 1.52
Appointment altered	1.52 *	1.06, 2.17
Telemedicine obstetrician ^†^	1.28	0.93, 1.76
Limited access to outdoor space ^†^	2.21 ***	1.57, 3.11
Compliance with the safety rules and restrictions	1.12	0.67, 1.88
	*R^2^* = 0.08	

* *p* < 0.05, ** *p* < 0.01, *** *p* < 0.001. AOR—Adjusted Odds Ratio; CI—Confidence Interval. ^†^ Women who reported below average or average financial status were grouped together. Women who reported perceived risk of having COVID-19 and those who were unsure were grouped together. Women who reported no telemedicine obstetrician during COVID-19 and those who reported it before the pandemic were grouped together. Women who reported sometimes or rarely having access to outdoor space were grouped together.

## Data Availability

The datasets used and/or analyzed during the current study are available from the corresponding author upon request.
